# Stronger Minds, Better Lives: Exercise Self-Efficacy and Resilience as Serial Mediators in Oncology Nurses

**DOI:** 10.3390/healthcare14101416

**Published:** 2026-05-21

**Authors:** Gülay Oyur Çelik, Mehmet Behzat Turan, Melih Balyan, Barış Karaoğlu, Osman Pepe, İbrahim Dalbudak, Bilgehan Pepe, Seda Evyapan Aydin, Mustafa Kara, Şıhmehmet Yiğit

**Affiliations:** 1 Faculty of Health Sciences, Izmir Katip Celebi University, Izmir 35620, Türkiye; 2 Faculty of Sports Sciences, Erciyes University, Kayseri 38039, Türkiye; 3 Faculty of Sports Sciences, Ege University, Izmir 35100, Türkiye; 4 Faculty of Sports Sciences, Bingol University, Bingol 12000, Türkiye; 5 Faculty of Sports Sciences, Süleyman Demirel University, Isparta 32260, Türkiye; 6 Faculty of Sports Sciences, Uşak University, Usak 64200, Türkiye; sihmehmet.yigit@usak.edu.tr; 7 Department of Exercise and Sports Sciences for People with Disabilities, Gelişim University, Istanbul 34310, Türkiye; 8 Institute of Graduate Studies, Uşak University, Usak 64200, Türkiye; 9 Institute of Health Sciences, Celal Bayar University, Manisa 45140, Türkiye

**Keywords:** mental health continuum, quality of life, exercise self-efficacy, psychological resilience, serial mediation, oncology nurses

## Abstract

**Background**: Oncology nurses are highly vulnerable to impaired mental health and reduced quality of life due to the emotionally demanding nature of their work. Although mental health is a well-established determinant of quality of life, the mechanisms underlying this relationship remain insufficiently understood. **Objective**: This study examined the effect of the mental health continuum on quality of life among oncology nurses and tested the serial mediating roles of exercise self-efficacy and psychological resilience. **Methods**: A cross-sectional design was conducted with 604 oncology nurses in Türkiye. Data were collected using the Mental Health Continuum—Short Form, the Exercise Self-Efficacy Scale, the Psychological Resilience Scale, and the WHOQOL-BREF. Serial mediation analysis was performed using PROCESS Model 6 with 5000 bootstrap resamples. **Results**: The mental health continuum had a significant positive effect on exercise self-efficacy (a1 = 0.08, *p* < 0.001) and psychological resilience (a2 = 0.05, *p* < 0.001). Exercise self-efficacy significantly predicted psychological resilience (d1 = 0.51, *p* < 0.001). Both exercise self-efficacy (b1 = 0.88, *p* < 0.001) and psychological resilience (b2 = 1.60, *p* < 0.001) were significant predictors of quality of life. The direct effect of the mental health continuum on quality of life remained significant (c′ = 0.65, *p* < 0.001), indicating partial mediation. Bootstrap results further confirmed that all indirect effects were statistically significant, as their 95% confidence intervals did not include zero. **Conclusions**: Quality of life is influenced not only by mental health but also by sequential cognitive and adaptive processes. Interventions targeting exercise self-efficacy and psychological resilience may enhance well-being among oncology nurses.

## 1. Introduction

Healthcare systems rely not only on technological advancements and organizational capacity but also on the psychological well-being of healthcare professionals. Among these professionals, oncology nurses are particularly vulnerable due to the emotionally demanding nature of their work, including continuous exposure to patient suffering, uncertainty, and end-of-life care. These conditions place oncology nurses at increased risk of chronic stress, emotional exhaustion, and burnout, which may negatively affect both their well-being and the quality of patient care [1,2,3]. Recent evidence further underscores that oncology nurses experience significantly higher levels of emotional exhaustion, compassion fatigue, and reduced well-being than other healthcare professionals due to continuous exposure to suffering and end-of-life care [4,5,6]. These occupational stressors not only affect psychological functioning but also undermine overall quality of life, reinforcing the need to examine underlying psychosocial mechanisms in this population. Accordingly, understanding the psychological mechanisms underlying oncology nurses’ well-being is critical.

Mental health, conceptualized as a continuum encompassing emotional, psychological, and social well-being, represents a central determinant of quality of life [7] rather than merely reflecting the absence of mental illness. Mental health functions as a foundational psychological resource that shapes individuals’ coping capacity, motivation, and behavioral responses to stress [8,9]. In high-stress professional settings such as oncology nursing, stronger mental health is associated with better adaptive functioning and improved quality-of-life outcomes [1].

However, the relationship between mental health and quality of life may not operate solely through direct pathways. Emerging evidence suggests that cognitive and adaptive mechanisms play a key role in this relationship. In this context, exercise self-efficacy reflects individuals’ perceived capability to initiate and maintain physical activity, serving as an important cognitive mechanism influencing behavior and coping processes [10,11,12]. Individuals with higher levels of mental health tend to report stronger self-efficacy beliefs due to enhanced psychological energy, positive affect, and self-regulation capacity [13,14].

Self-efficacy also contributes to the development of psychological resilience, defined as the capacity to adapt to and recover from stress [15]. Individuals with higher self-efficacy are more likely to adopt effective coping strategies and sustain adaptive functioning under challenging conditions, thereby strengthening resilience [16]. Within the Theory of Planned Behavior, perceived behavioral control further supports this process by enabling individuals to manage stress more effectively [17]. In healthcare contexts, resilience has been consistently linked to reduced burnout and improved quality of life [1,2,18].

Quality of life is a multidimensional construct reflecting individuals’ overall well-being across physical, psychological, and social domains [19]. Previous research demonstrates that higher levels of psychological resilience are associated with lower stress and enhanced quality of life [20,21,22]. These findings suggest that mental health may influence quality of life through a multi-stage psychological process involving both cognitive (self-efficacy) and adaptive (resilience) mechanisms.

Despite increasing interest in these variables, studies examining mental health, exercise self-efficacy, psychological resilience, and quality of life within a single process-oriented model remain limited. Moreover, research employing serial mediation approaches, particularly among high-risk groups such as oncology nurses, is scarce [23]. Addressing this gap, the present study investigates the serial mediating roles of exercise self-efficacy and psychological resilience in the relationship between mental health and quality of life among oncology nurses.

While previous studies have examined mental health, self-efficacy, and resilience separately, the present study contributes to the literature by proposing and testing a serial mediation model that integrates these constructs within a unified framework among oncology nurses.

### 1.1. Mental Health Continuum

Mental health has traditionally been conceptualized as the absence of psychopathology; however, contemporary perspectives emphasize a more comprehensive and dynamic understanding. Within this framework, mental health is defined as a multidimensional construct encompassing emotional, psychological, and social well-being. The mental health continuum model further conceptualizes mental health along a spectrum ranging from languishing to flourishing, highlighting that individuals may experience varying levels of well-being regardless of the presence of psychological distress [24].

This multidimensional perspective is particularly relevant in high-stress professional contexts such as healthcare. For oncology nurses, mental health plays a critical role in shaping coping capacity, motivation, and overall occupational functioning. Empirical evidence indicates that lower levels of mental health are associated with higher burnout and reduced quality of care, whereas higher levels contribute to improved performance and well-being [1,3,25].

Recent research increasingly conceptualizes mental health not merely as an outcome, but as a foundational psychological resource that influences individuals’ cognitive and behavioral processes. Individuals with higher mental health tend to exhibit greater psychological energy, positive affect, and self-regulation capacity, which in turn strengthen self-efficacy beliefs and adaptive functioning [8,9,11]. However, the influence of mental health on well-being outcomes may not operate solely through direct effects. Instead, emerging evidence suggests that this relationship is shaped by underlying psychological mechanisms, including cognitive appraisals and coping capacities [20].

Accordingly, the present study conceptualizes mental health as the primary antecedent (X) that initiates a multi-stage psychological process. Specifically, mental health is proposed to influence exercise self-efficacy, a cognitive mechanism, and psychological resilience, an adaptive capacity, both of which contribute to quality of life. This process-oriented perspective provides a more comprehensive explanation of how internal psychological resources are translated into well-being outcomes.

### 1.2. Exercise Self-Efficacy

Self-efficacy is a key cognitive mechanism through which mental health influences individuals’ behavioral tendencies. Defined as individuals’ perceived capability to initiate and maintain physical activity despite potential barriers, exercise self-efficacy plays a central role in regulating motivation, behavior, and responses to environmental demands within the framework of Social Cognitive Theory [11,12]. Beyond its role in promoting physical activity, self-efficacy also shapes how individuals appraise and cope with stress. Individuals with higher self-efficacy are more likely to perceive challenges as manageable, maintain motivation, and persist in the face of difficulties [13].

Although the relationship between mental health and self-efficacy is often considered bidirectional, recent perspectives increasingly emphasize the role of mental health as a foundational antecedent. Individuals with higher levels of mental health tend to exhibit greater psychological energy, positive affect, and self-regulation capacity, which contribute to stronger perceptions of self-efficacy [8,9,11]. This perspective is particularly relevant in high-stress contexts such as healthcare, where psychological resources are critical for maintaining adaptive functioning.

Importantly, exercise self-efficacy extends beyond a belief system and functions as a mechanism that shapes higher-order adaptive processes. By influencing cognitive appraisals and coping strategies, self-efficacy facilitates individuals’ ability to manage stress effectively. Individuals with stronger self-efficacy beliefs are more likely to engage in problem-focused coping and sustain adaptive efforts, which, over time, contribute to the development of psychological resilience [16].

Accordingly, in the present study, exercise self-efficacy is conceptualized as a critical intermediary mechanism (M1) linking mental health to psychological resilience. This positioning reflects a process-oriented perspective in which mental health shapes cognitive evaluations, which in turn are translated into adaptive capacity.

### 1.3. Psychological Resilience

Psychological resilience represents an adaptive capacity that enables individuals to cope effectively with stress and recover from adverse experiences [15]. Rather than a fixed trait, resilience is conceptualized as a dynamic and developmental process shaped by cognitive appraisals, emotional regulation, and coping strategies [26]. From this perspective, how individuals interpret and respond to stressors plays a central role in the development and maintenance of resilience.

Within this framework, self-efficacy serves as an important cognitive mechanism that supports resilience. According to Social Cognitive Theory, individuals with higher self-efficacy are more likely to perceive challenges as manageable, sustain effort, and adopt adaptive coping strategies [11]. Empirical evidence further indicates that stronger self-efficacy beliefs facilitate adaptive responses to stress, thereby contributing to the development of psychological resilience over time [16,27]. In high-demand professional settings such as healthcare, these processes are particularly critical, as individuals must continuously adapt to emotionally and physically challenging conditions [3].

Although resilience is influenced by contextual factors, including organizational climate and social support, it remains a central mechanism linking cognitive processes to well-being outcomes. In line with the Theory of Planned Behavior, perceived behavioral control, closely related to self-efficacy, further enhances individuals’ capacity to manage stress effectively [17]. Through repeated adaptive responses, these processes strengthen resilience and support sustained psychological functioning [22,28].

Importantly, psychological resilience has been consistently associated with improved quality of life. Individuals with higher resilience tend to report lower levels of stress and burnout, as well as better psychological functioning and overall well-being [2,20,22]. Recent research also highlights the protective role of resilience in maintaining well-being among healthcare professionals exposed to high levels of occupational stress [29]. These findings suggest that resilience functions as a key adaptive mechanism through which internal psychological resources are translated into tangible life outcomes.

Accordingly, in the present study, psychological resilience is conceptualized as a sequential mediator (M2) linking exercise self-efficacy to quality of life. This positioning reflects a process-oriented framework in which cognitive beliefs (self-efficacy) are transformed into adaptive capacity (resilience), ultimately contributing to enhanced well-being.

### 1.4. Quality of Life

Quality of life is widely conceptualized as a multidimensional construct reflecting individuals’ overall well-being across physical, psychological, and social domains [19]. Beyond the absence of illness, it encompasses subjective well-being, functional capacity, and life satisfaction, emphasizing individuals’ evaluations of their living conditions [30]. Accordingly, quality of life serves as a comprehensive indicator of how individuals adapt to environmental demands and utilize their internal psychological resources.

A substantial body of research demonstrates that quality of life is closely associated with key psychological factors, particularly psychological resilience. Individuals with higher resilience report lower perceived stress, better psychological adjustment, and enhanced quality of life [20,21,22]. In high-stress occupational settings such as healthcare, these relationships become even more pronounced, as psychological resources play a critical role in maintaining well-being and functional performance.

Empirical evidence further indicates that a lower quality of life among healthcare professionals is associated with increased burnout, job dissatisfaction, and reduced performance. In contrast, a higher quality of life contributes to improved psychological functioning and quality of care [1,2,3]. Within this context, quality of life can be conceptualized as the primary outcome through which internal psychological processes are manifested.

From a process-oriented perspective, quality of life is shaped through a multi-stage mechanism. Mental health functions as a foundational resource influencing individuals’ emotional and cognitive functioning; exercise self-efficacy reflects perceived behavioral competence; and psychological resilience represents adaptive capacity in response to stress. Together, these processes form a sequential pathway through which internal resources are translated into well-being outcomes.

Accordingly, examining these relationships within a serial mediation framework provides a comprehensive understanding of how cognitive and adaptive processes jointly contribute to quality of life [23].

### 1.5. The Present Study

Although the relationships among mental health, self-efficacy, psychological resilience, and quality of life have been widely examined, prior research has largely relied on independent or direct-effect models. In particular, mental health has often been conceptualized as an outcome variable, whereas its role as a foundational antecedent shaping cognitive and adaptive processes has received limited attention [7,8]. This limitation restricts a comprehensive understanding of the mechanisms through which mental health influences well-being outcomes.

Moreover, although self-efficacy and psychological resilience are recognized as key psychological resources, they have predominantly been examined as parallel predictors rather than as components of a sequential process. From a theoretical perspective, self-efficacy reflects individuals’ cognitive beliefs regarding their capability to perform behaviors. In contrast, psychological resilience represents the adaptive transformation of these beliefs into sustained coping capacity over time [26]. Accordingly, these constructs are better understood as part of a directional, multi-stage mechanism rather than as independent factors.

Similarly, while quality of life is widely acknowledged as a fundamental indicator of well-being, the psychological processes underlying its formation remain insufficiently explored. Existing research has primarily focused on environmental or individual determinants, with limited attention to integrative, process-oriented models that incorporate both cognitive and adaptive mechanisms [1,19].

To address these gaps, the present study adopts a process-oriented framework. It proposes a serial mediation model in which mental health (X) influences quality of life (Y) through exercise self-efficacy (M1) and psychological resilience (M2). Grounded in Social Cognitive Theory [11] and the Theory of Planned Behavior [17], the model conceptualizes mental health as a foundational resource that shapes individuals’ cognitive evaluations (self-efficacy), which, in turn, facilitates adaptive capacity (resilience) and ultimately contributes to improved quality of life.

This study focuses on oncology nurses, a high-risk professional group exposed to persistent emotional and occupational stressors. Despite their vulnerability, research examining their psychological functioning through multivariate and mechanism-based models remains limited [1,2].

Accordingly, the primary objective of this study is to examine the serial mediating roles of exercise self-efficacy and psychological resilience in the relationship between mental health and quality of life among oncology nurses. By integrating cognitive and adaptive mechanisms within a unified framework, this study aims to provide a more comprehensive explanation of how internal psychological resources are translated into well-being outcomes.

Accordingly, the proposed model aims to examine not only the relationships among the study variables but also the underlying multi-stage psychological mechanisms linking mental health to quality of life.

Based on the theoretical framework, the following hypotheses were formulated:

**Direct Effects**

**H1.**  *Mental health continuum positively predicts quality of life.*

**H2.**  *Mental health continuum positively predicts exercise self-efficacy.*

**H3.**  *Exercise self-efficacy positively predicts psychological resilience.*

**H4.**  *Psychological resilience positively predicts quality of life.*

**Mediating Effects**

**H5.**  *Exercise self-efficacy mediates the relationship between mental health continuum and quality of life.*

**H6.**  *Psychological resilience mediates the relationship between the mental health continuum and quality of life.*

**Serial Mediation Effect**

**H7.**  *Exercise self-efficacy and psychological resilience sequentially mediate the relationship between the mental health continuum and quality of life.*

### 1.6. Conceptual Model and Theoretical Framework

This study is grounded in Social Cognitive Theory (SCT) and the Theory of Planned Behavior (TPB) to explain the relationships among mental health, exercise self-efficacy, psychological resilience, and quality of life. These complementary frameworks provide a process-oriented perspective, illustrating how emotional states translate into cognitive beliefs, evolve into adaptive capacities, and ultimately shape well-being.

According to SCT, behavior is shaped through the interaction of cognitive, emotional, and environmental factors, with self-efficacy representing a central mechanism regulating motivation, effort, and persistence [11]. Importantly, internal psychological states such as emotional well-being and stress levels influence individuals’ perceptions of competence [9,11,31]. Accordingly, mental health can be conceptualized as a foundational psychological resource that shapes cognitive evaluations and perceived behavioral competence.

Extending this perspective, the present study positions mental health as an antecedent rather than an outcome variable. Drawing on the mental health continuum model [7], individuals with higher mental health exhibit greater psychological energy, positive affect, and self-regulation capacity, which contribute to stronger self-efficacy beliefs [8]. This mechanism is further supported by TPB, in which perceived behavioral control closely aligned with self-efficacy guides behavioral responses and coping strategies [17]. Together, SCT and TPB explain how internal psychological states are translated into cognitive beliefs and adaptive behavioral processes.

The next stage in this process is psychological resilience, defined as the capacity to adapt to and recover from stress [15]. Self-efficacy and resilience form a sequential relationship: self-efficacy reflects individuals’ cognitive appraisals of their ability to manage challenges, whereas resilience represents the transformation of these appraisals into sustained adaptive capacity [26]. Accordingly, these constructs should be understood as components of a directional, multi-stage process rather than parallel predictors [3].

Psychological resilience plays a key role in shaping quality of life, a multidimensional indicator of overall well-being [19]. Individuals with higher resilience report lower stress and better psychological adjustment, which contribute to enhanced quality of life [20,21,22]. In healthcare contexts, resilience is also associated with reduced burnout and improved professional functioning [1,2].

Based on this framework, the present study proposes a serial mediation model in which mental health (X) influences exercise self-efficacy (M1), which in turn enhances psychological resilience (M2), ultimately leading to improved quality of life (Y). This model reflects a coherent pathway through which emotional, cognitive, and adaptive processes jointly shape well-being.

This study contributes to the literature in three ways. First, it reconceptualizes mental health as a foundational antecedent rather than merely an outcome. Second, it advances a process-oriented perspective by examining self-efficacy and resilience as sequential mechanisms. Third, it highlights the importance of targeting emotional, cognitive, and adaptive processes in interventions to improve well-being.

In conclusion, this framework demonstrates that mental health, self-efficacy, and psychological resilience operate in an interconnected manner to shape quality of life. Accordingly, testing these relationships through a serial mediation model provides a theoretically grounded approach to understanding multi-stage well-being mechanisms [23].

## 2. Methods

### 2.1. Study Design

This study employed a cross-sectional research design to examine the relationships among mental health, exercise self-efficacy, psychological resilience, and quality of life among oncology nurses.

This study employed a correlational survey design to examine the relationships among the study variables. The correlational survey model is a non-experimental research approach that aims to determine the degree of covariation between two or more variables and to assess the presence, direction, and strength of relationships among them [32,33]. This approach enables the identification of potential predictive relationships without manipulating variables. Within this framework, the study aimed to investigate the relationships among oncology nurses’ levels of mental health continuum, exercise self-efficacy, psychological resilience, and quality of life [34]. In addition, the study examined the serial mediating roles of exercise self-efficacy and psychological resilience in the relationship between the mental health continuum and quality of life using a process-oriented analytical approach [35].

**Figure 1:**
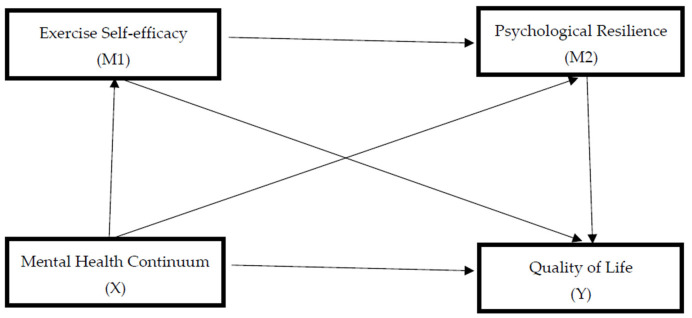
The serial mediation model of mental health continuum, exercise self-efficacy, psychological resilience, and quality of life.

Accordingly, the mental health continuum (X) was specified as the independent variable and quality of life (Y) as the dependent variable. Exercise self-efficacy (M1) and psychological resilience (M2) were modeled as sequential mediators explaining the effect of the mental health continuum on quality of life. For clarity, the hypothesized relationships among variables are also presented schematically in Figure 1, illustrating the sequential pathway from mental health to quality of life through exercise self-efficacy and psychological resilience.

As illustrated in Figure 1, the serial mediating roles of exercise self-efficacy and psychological resilience in the relationship between the independent and dependent variables were examined using the PROCESS Macro Model 6 [35]. This analysis enabled simultaneous testing of the mediating and sequential pathways proposed in the study model.

### 2.2. Study Sample Size Determination

In the present study, the required sample size was determined via Monte Carlo simulation to ensure adequate statistical power to detect indirect effects in a serial multiple mediation model. This approach is considered more appropriate than traditional analytical methods, particularly in complex mediation models involving multiple sequential mediators, as it allows direct estimation of the sampling distribution of indirect effects [36,37]. Additionally, it addresses the non-normal distribution of indirect effects and provides more accurate power estimates [38]. The serial mediation model was specified as follows: mental health continuum (X) → exercise self-efficacy (M1) → psychological resilience (M2) → quality of life (Y). The primary parameter of interest was the serial indirect effect (a_1_ × d_21_ × b_2_). For power estimation, standardized path coefficients of β ≈ = 0.30 were assumed for each structural path, reflecting a moderate effect size commonly reported in the literature [37,38]. Based on 2000 Monte Carlo replications, statistical power was defined as the proportion of simulations in which the 95% confidence interval for the indirect effect did not include zero. The results indicated that a minimum sample size of approximately N ≈ 400 is sufficient to detect the serial indirect effect with at least 80% power, consistent with methodological recommendations [36,37]. The final sample of the present study consisted of N = 604 participants, exceeding the minimum required sample size. This indicates that the study had high statistical power to detect both direct and indirect effects. Consequently, parameter estimates are expected to be more stable and less affected by sampling error. Accordingly, the sample size of N = 604 is considered sufficient for testing the proposed serial mediation model. It provides robust estimation of the relationships among Mental Health Continuum, Exercise Self-Efficacy, Psychological Resilience, and Quality of Life.

### 2.3. Study Procedure

Data were collected using an online survey distributed to oncology nurses working in public and private healthcare institutions. Participation was voluntary, and informed consent was obtained from all participants prior to data collection.

#### 2.3.1. Participants

The target population of this study comprised nurses working in hospitals across Türkiye as of 2025. The inclusion criteria for participation were defined as follows: (i) being 18 years of age or older, (ii) having at least five years of professional experience as an oncology nurse, (iii) currently working in an oncology unit within their affiliated hospital, and (iv) providing voluntary informed consent to participate in the study. Nurses working in non-oncology units were excluded from the study.

A convenience sampling strategy, a commonly used non-probability sampling method, was employed to recruit participants. This approach allows researchers to collect data from readily accessible individuals who are willing to participate and is widely used in social science research [39].

Data were collected through an online questionnaire administered via Google Forms. To facilitate data collection, administrators of public and private hospitals across seven geographical regions of Türkiye were contacted, and the survey link was distributed electronically to nurses working in oncology units. At the end of the data collection process, a total of 626 nurses working in oncology units of public and private hospitals across Türkiye were initially included in the study. Following preliminary data screening, 10 responses containing incomplete or erroneous entries were excluded from the analysis. In addition, 12 cases were removed via outlier analysis to ensure the dataset’s robustness. Data collection was conducted within a defined time frame (15 September–15 October 2025). After completing all data-cleaning procedures, the final sample comprised 604 oncology nurses employed in public and private hospitals across Türkiye.

#### 2.3.2. Data Collection Tools

In this study, data were collected through an online survey administered via Google Forms. Participants were first asked to complete a demographic information form developed by the researchers. In addition, standardized measurement instruments validated in the literature were used to assess the mental health continuum, exercise self-efficacy, psychological resilience, and quality of life. Before participation, all respondents were required to complete an informed consent form confirming their voluntary participation in the study.

##### Personal Information Form

A researcher-developed personal information form was used to collect participants’ demographic and professional characteristics. The form consisted of five items assessing age, gender, hospital of employment, perceived economic status, and years of professional experience.

Table 1 presents the demographic characteristics of the participants. Accordingly, 39.4% of the participants were under 30 years old, 26.2% were between 31 and 40 years old, and 34.4% were aged 41 years and above. In terms of gender distribution, the majority of the sample (92.9%) consisted of male participants, while the remaining 7.1% were female. Regarding workplace characteristics, 54.1% of the participants were employed in state hospitals, whereas 45.9% worked in private hospitals. Regarding perceived economic status, 15.2% reported low income, 77.2% reported middle income, and 7.6% reported high income. Regarding professional experience, 50.5% of participants had 5–10 years, 26.5% had 11–20 years, and 23.0% had 21+ years.

##### Mental Health Continuum—Short Form (MHC-SF)

The Mental Health Continuum—Short Form (MHC-SF), a self-report instrument designed to assess individuals’ emotional, psychological, and social well-being, was developed by Keyes et al. [40]. The scale consists of 14 items across three sub-dimensions: emotional well-being (items 1–3), social well-being (items 4–8), and psychological well-being (items 9–14). Participants are asked to respond to the core question, “How often did you experience the following feelings during the past month?” using a 6-point Likert-type scale ranging from 0 (never) to 5 (every day). Total scores range from 0 to 70, with higher scores indicating better mental health. The scale does not include any reverse-coded items, and the total score is obtained by summing responses across all items. The Turkish adaptation of the scale was conducted by Demirci and Akın [41]. The reported internal consistency coefficients (Cronbach’s alpha) for the Turkish version were 0.84 for emotional well-being, 0.78 for social well-being, and 0.85 for psychological well-being, with an overall reliability coefficient of 0.90. Example items from the scale include “I felt happy” and “I felt that my life had a sense of direction or meaning.”

##### Exercise Self-Efficacy Scale (ESES)

The Exercise Self-Efficacy Scale, originally developed by Marcus et al. [42] and adapted into Turkish by Ay and Temel [43], was used to assess individuals’ perceived competence in maintaining exercise behavior under various conditions. The scale has a unidimensional structure and is designed to assess individuals’ self-efficacy beliefs regarding engaging in physical activity despite potential barriers. The instrument consists of six items rated on a 5-point Likert-type scale ranging from 1 (not at all confident) to 5 (completely confident). Total scores range from 6 to 30, with higher scores indicating greater levels of exercise self-efficacy. The Turkish adaptation study reported strong psychometric properties, with a high internal consistency coefficient (Cronbach’s alpha = 0.90) and satisfactory test–retest reliability [43]. Example items include “I exercise even when I am under a lot of stress” and “I exercise even when I do not have time.”

##### Psychological Resilience Scale—Brief Form (BRS-BREF)

Psychological Resilience Scale (BRS), developed by Smith et al. [44], was used to assess individuals’ ability to recover from stress and adapt effectively to challenging situations. The Turkish adaptation, along with validity and reliability analyses, was conducted by Doğan [45]. The scale has a unidimensional structure and consists of six items designed to measure individuals’ levels of psychological resilience [44,45]. Items are rated on a 5-point Likert-type scale ranging from 1 (strongly disagree) to 5 (strongly agree). Three items (items 2, 4, and 6) are reverse-coded. Total scores are calculated by averaging item responses, with higher scores indicating greater psychological resilience. The original scale demonstrated good internal consistency, with Cronbach’s alpha values ranging between 0.80 and 0.91, while the Turkish adaptation study reported an internal consistency coefficient of α = 0.83 [44,45]. An example item from the scale is: “I tend to bounce back quickly after hard times.”

##### WHO Quality of Life Scale—Brief Form (WHOQOL-BREF)

The World Health Organization Quality of Life Scale—Brief Form (WHOQOL-BREF), developed by the WHOQOL Group [46], was used to assess individuals’ quality of life within a multidimensional framework. While the original WHOQOL instrument consists of 100 items, the abbreviated WHOQOL-BREF includes 26 items. The scale evaluates four core domains: physical health, psychological health, social relationships, and environmental conditions [46]. The Turkish adaptation of the WHOQOL-BREF was conducted by Eser et al. [47]. In the adaptation study, the internal consistency coefficients (Cronbach’s alpha) were reported as 0.83 for physical health, 0.66 for psychological health, 0.53 for social relationships, and 0.73 for the environmental domain. The overall reliability coefficient for the total scale was reported as α = 0.86 [47].

Items are rated on a Likert-type scale, and higher scores indicate better perceived quality of life. Domain scores can be calculated separately; however, in the present study, an overall quality-of-life score was used for analysis. Example items include “How would you rate your quality of life?” and “How much do you enjoy your life?”

### 2.4. Measures

#### 2.4.1. Common Method Bias

In this study, the use of a single data source and self-report measures may introduce the risk of common method bias (CMB). To assess this, Harman’s single-factor test was conducted, indicating that a single factor accounted for less than 50% of the total variance. This suggests that CMB is unlikely to have substantially affected the results [48]. Additionally, procedural remedies were applied, including ensuring participant anonymity and emphasizing that there were no right or wrong answers, to reduce social desirability and evaluation bias.

To further examine common method variance, a Common Latent Factor (CLF) analysis was performed. The inclusion of the latent factor did not result in substantial changes in standardized regression coefficients (Δ < 0.20), supporting the conclusion that common method variance does not pose a serious threat to the validity of the findings [48].

Although the study employed a cross-sectional design, the proposed model is grounded in established theoretical frameworks, namely Social Cognitive Theory and the Theory of Planned Behavior. Furthermore, the use of serial multiple mediation analysis with bootstrapping enhances the robustness of the findings by enabling the estimation of indirect effects from empirical sampling distributions [35]. Nevertheless, future studies using longitudinal and experimental designs are recommended to establish causal relationships more clearly. The internal consistency coefficients (Cronbach’s alpha) derived from the participants’ responses are presented in Table 2.

Table 2 shows that the Cronbach’s Alpha vaLues indicate internal consistency coefficients of 0.93 for the Mental Health Continuum—Short Form, 0.82 for the Exercise Self-efficacy, 0.85 for the Psychological Resilience-Short Form, and 0.95 for the World Health Organization Quality of Life. These values indicate that the data provided by participants on these scales possess an acceptable level of internal consistency.

#### 2.4.2. Discriminant Validity Assessment

Discriminant validity was assessed to determine whether the study constructs are conceptually distinct. The Fornell–Larcker criterion and the Heterotrait–Monotrait ratio (HTMT) were employed [49,50].

The results showed that the square roots of the average variance extracted (AVE) for all constructs exceeded the corresponding inter-construct correlations, satisfying the Fornell–Larcker criterion. In addition, all HTMT values were below the recommended threshold of 0.85, further supporting discriminant validity [50]. Inter-construct correlations were also below 0.85, indicating no problematic overlap among variables [51].

Overall, these findings confirm that Mental Health Continuum, Exercise Self-Efficacy, Psychological Resilience, and Quality of Life are empirically distinct constructs and that the measurement model is suitable for subsequent structural analyses.

#### 2.4.3. Robustness Analysis Using Knowledge Dimension

To assess the robustness of the findings, additional analyses were conducted using alternative model specifications. Specifically, the sub-dimensions of the mental health continuum (emotional, psychological, and social well-being) were entered into the model separately to evaluate the stability of the serial mediation structure across different components.

The results indicated that the serial mediating role of exercise self-efficacy and psychological resilience remained consistent in both direction and statistical significance across all model specifications. Furthermore, the confidence intervals of the indirect effects did not include zero, providing additional support for the stability of the mediation effects [23].

Overall, these findings demonstrate that the proposed model is robust and not dependent on a specific operationalization of mental health. The consistency of results across alternative specifications strengthens the reliability and credibility of the serial mediation model.

### 2.5. Statistical Analysis

The data were analyzed using IBM SPSS Statistics for Windows, Version 22.0 (IBM Corp., Armonk, NY, USA). A Monte Carlo Simulation was conducted to evaluate the adequacy of the sample size. Descriptive statistics, including frequency and percentage distributions, were calculated. To determine suitability for parametric tests, the normality of variable distributions was assessed using skewness and kurtosis. It has been determined that the skewness and kurtosis values for the data fall within ±2 [52]. Accordingly, Pearson Correlation analysis was used to determine the correlations between the variables. A Simple Linear Regression Analysis was performed to examine the effect of the independent variable on the dependent variable. To test the hypothesis of serial mediating roles for exercise self-efficacy and psychological resilience, the PROCESS macro developed by Hayes [35] was applied in SPSS. The Macro Model 6 option was used to examine the serial mediating effect. During this analysis, the Bootstrap method with 5000 resamples was selected. In this method, the values obtained in the 95% confidence interval must not include zero (0) values [35,53].

Given the cross-sectional nature of the data, the mediation model tested in this study reflects statistical associations rather than causal pathways. Therefore, the directionality of the relationships should be interpreted cautiously and within the bounds of theoretical plausibility [54].

## 3. Results

Descriptive statistics for the study variables are presented in Table 3. The mean scores were 28.32 (SD = 13.04) for Mental Health Continuum, 14.53 (SD = 3.93) for Exercise Self-Efficacy, 17.88 (SD = 4.76) for Psychological Resilience, and 82.87 (SD = 19.33) for Quality of Life (N = 604), indicating moderate levels across the constructs. Skewness (−0.27 to 0.19) and kurtosis (−1.10 to −0.53) values fell within acceptable ranges, supporting the assumption of normality [52,55]. These findings indicate that the distributions were approximately symmetrical and suitable for parametric analyses. Overall, the results confirm that the dataset meets the assumptions required for subsequent analyses.

An examination of Table 4, the results of the correlation analysis indicated that there were positive and statistically significant relationships among mental health continuum, exercise self-efficacy, psychological resilience, and quality of life (*p* < 0.01). Mental health continuum showed low-level positive correlations with exercise self-efficacy (r = 0.26) and psychological resilience (r = 0.24). It also showed a moderate-to-high positive correlation with quality of life (r = 0.58). Exercise self-efficacy was moderately and positively correlated with psychological resilience (r = 0.46) and quality of life (r = 0.47). In addition, psychological resilience demonstrated a moderate-to-high positive relationship with quality of life (r = 0.58). Overall, these findings suggest that all variables are positively related to each other, with quality of life showing particularly strong associations with mental health continuum and psychological resilience.

As shown in Table 5, the regression model reveals a significant relationship between the nurses’ mental health continuum and quality of life (F(1, 602) = 300.43, *p* < 0.001). According to the t-test results on the significance of the regression coefficient, the quality of life significantly predicts the mental health continuum (t = 17.33, *p* < 0.001). This model explains 33% of the variance in the participants’ quality of life (R^2^ = 0.33, *p* < 0.001).

Multicollinearity among the Predictor variables was assessed using tolerance and Variance Inflation Factor (VIF) values (Table 6). The results indicated acceptable tolerance and VIF values for all variables: Exercise Self-Efficacy (tolerance = 0.768; VIF = 1.302), Psychological Resilience (tolerance = 0.775; VIF = 1.291), and Mental Health Continuum (tolerance = 0.915; VIF = 1.092). All tolerance values exceeded 0.10, and VIF values were well below both the standard threshold of 10 and the more conservative threshold of 5, indicating no multicollinearity concerns [55,56]. These findings suggest that the predictor variables are not highly correlated and contribute independently to the model [57].

Overall, the assumption of no multicollinearity is satisfied, supporting the reliability of the regression analyses.

Cook’s Distance values (Table 7) were examined to assess the influence of individual observations on the regression model [58]. The values ranged from 0.000 to 0.088 (M = 0.003, SD = 0.010; N = 604).

All values were well below the commonly accepted thresholds of 1.00 and the more conservative criterion of 0.50, indicating the absence of influential observations [55,56,57].

Overall, the results suggest that no single case exerted a disproportionate influence on the model, supporting the robustness and reliability of the regression analyses.

The regression results (Table 8) indicate a strong relationship between the predictor variables and the dependent variable (R = 0.751). The model explains 56.4% of the variance (R^2^ = 0.564), with a similar adjusted value (Adjusted R^2^ = 0.562), suggesting no overfitting and good generalizability [56,59].

The overall model was statistically significant, F(3, 600) = 258.575, *p* < 0.001, indicating that the predictors jointly contribute to the outcome variable [55]. The standard error of the estimate (SE = 12.80) suggests acceptable prediction accuracy.

Overall, the findings demonstrate that the model is statistically significant, explanatory, and robust.

The ANOVA results presented in Table 9 indicate that the regression model is statistically significant, F(3, 600) = 258.575, *p* < 0.001. This finding demonstrates that the predictor variables collectively explain a significant proportion of the variance in quality of life.

In terms of variance decomposition, the model accounts for 127,047.420 of the total sum of squares (SS = 225,314.667), while 98,267.247 is attributed to residual variance. The regression mean square (MS = 42,349.140) is substantially higher than the residual mean square (MS = 163.779), further supporting the model’s explanatory power.

Overall, these results confirm that the regression model provides a good fit to the data and that the predictors jointly contribute significantly to explaining quality of life.

The regression coefficients in Table 10 indicate that all predictor variables had positive, statistically significant effects on quality of life (*p* < 0.001).

Exercise Self-Efficacy was a significant predictor (B = 0.878, β = 0.178, t = 5.795), while Psychological Resilience showed a stronger effect (B = 1.597, β = 0.393, t = 12.836). Mental Health Continuum emerged as the strongest predictor (B = 0.648, β = 0.437, t = 15.510), based on the highest standardized coefficient. Overall, the findings indicate that all predictors significantly contribute to quality of life. All reported effects were statistically significant (*p* < 0.001), indicating strong evidence against the null hypothesis. In addition, the bootstrap confidence intervals for the indirect effects did not include zero, further confirming the mediation pathways’ statistical significance.

**Figure 2:**
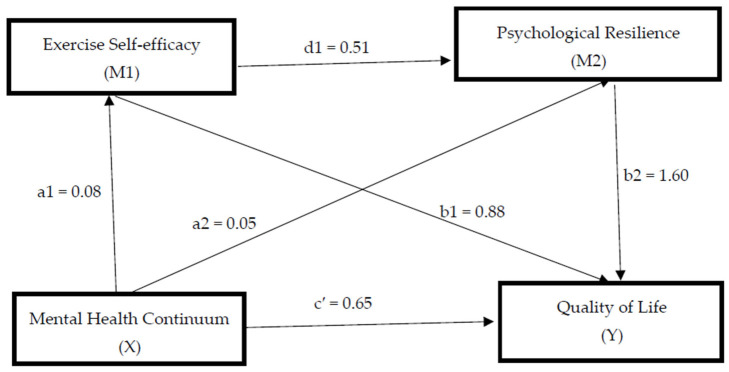
The serial mediation association between Exercise self-efficacy and psychological resilience. Standardized coefficients are reported. c′ = direct effect.

When Table 11 examined, Results indicated that the Mental Health Continuum variable had a positive and statistically significant effect on Exercise Self-efficacy (path a1) (a1 = 0.08, t = 6.50, *p* < 0.01). When the effect of Exercise Self-efficacy variable had a positive and significant effect on Quality of Life (path b1), (b1 = 0.88, t = 5.80, *p* < 0.01). It was also determined that the Mental Health Continuum variable had a positive and statistically significant effect on psychological resilience (path a2) (a2 = 0.05, t = 3.53, *p* < 0.01). When the effect of psychological resilience variable had a positive and significant effect on Quality of Life (path b2), (b2 = 1.60, t = 12.84, *p* < 0.01). In addition, Results indicated that the Exercise Self-efficacy variable had a positive and statistically significant effect on Psychological resilience (path d1) (d1 = 0.51, t = 11.41, *p* < 0.01). On the other hand, when the direct effect of Mental Health Continuum on Quality of Life (path c′) was analyzed, Results indicated that this effect was positive and statistically significant (c′ = 0.65, t = 15.51, *p* < 0.01). Figure 2 shows the serial mediation association between exercise self-efficacy and psychological resilience. In this model, paths a1 and a2 represent the effects of mental health on the mediators, path d1 represents the effect of exercise self-efficacy on psychological resilience, paths b1 and b2 represent the effects of the mediators on quality of life, and c′ represents the direct effect of mental health on quality of life after controlling for mediators.

As illustrated in Figure 2, model 6, a serial multiple mediation model, was used. The model contains two mediating variables, three indirect effects, and one direct effect. These effects are as follows: The indirect effect of Mental Health Continuum on Quality of Life through Exercise Self-efficacy (a1b1), The indirect effect of Psychological resilience via Mental Health Continuum on Quality of Life (a2b2), The indirect effect of Mental Health Continuum on Quality of Life via Exercise Self-efficacy and Psychological resilience (a1d1b2), The sum of these three indirect effects represents the total indirect effect of Quality of Life (Serial mediation effect: a1b1 + a2b2 + a1d1b2).

As indicated in Table 12, the Mental Health Continuum has a positive, statistically significant effect on Quality of Life (B = 0.21, SE = 0.04, CI = [0.13; 0.30]). The first indirect effect is the influence of the Mental Health Continuum on Quality of Life via Exercise Self-efficacy. [Ind1= Mental Health Continuum → Exercise Self-efficacy → Quality of Life]. This indirect effect is statistically significant (B = 0.07, SH = 0.02, CI = [0.03; 0.11]). The second indirect effect is the impact of Mental Health Continuum on Quality of Life through Psychological resilience. [Ind 2= Mental Health Continuum → Psychological resilience → Quality of Life]. This indirect effect is statistically significant (B = 0.08, SH = 0.03, CI = [0.03; 0.15]). The third indirect effect is the serial effect of Mental Health Continuum on Quality of Life through Exercise Self-efficacy and Psychological resilience. [Ind 3= Mental Health Continuum → Exercise Self-efficacy → Psychological resilience → Quality of Life]. This indirect effect is statistically significant. (B = 0.06, SH = 0.02, CI = [0.04; 0.10]).

## 4. Discussion

This study examined the psychosocial mechanisms underlying the relationship between mental health and quality of life among oncology nurses. The findings demonstrate that this relationship is not merely direct but operates through sequential psychological processes involving exercise self-efficacy and psychological resilience. This supports the growing view that well-being outcomes are better explained by mechanism-based, process-oriented models rather than simple linear associations [1,23].

This supports the growing view that well-being outcomes are better explained by mechanism-based, process-oriented models rather than simple linear associations.

These findings are consistent with Social Cognitive Theory, which emphasizes the role of self-efficacy in regulating behavior and coping processes [11], and the Theory of Planned Behavior, which highlights perceived behavioral control as a determinant of adaptive responses [17]. The results demonstrate how mental health, as an emotional resource, is translated into cognitive beliefs and adaptive capacity, ultimately shaping quality of life.

Regarding the study hypotheses, the findings supported all proposed hypotheses. Mental health continuum positively predicted quality of life (H1) and exercise self-efficacy (H2). Exercise self-efficacy positively predicted psychological resilience (H3), while psychological resilience positively predicted quality of life (H4). In addition, exercise self-efficacy (H5) and psychological resilience (H6) significantly mediated the relationship between the mental health continuum and quality of life. Finally, the serial mediation pathway involving exercise self-efficacy and psychological resilience was also supported (H7).

A key finding is that the mental health continuum emerged as the strongest predictor of quality of life, reinforcing its role as a foundational psychological resource. This is consistent with the mental health continuum framework, which conceptualizes mental health as encompassing emotional, psychological, and social well-being [7,8]. In healthcare contexts, particularly among oncology nurses, mental health has been consistently linked to reduced burnout and enhanced functioning [1,29]. However, the present findings extend this literature by demonstrating that the influence of mental health is not solely direct but is transmitted through intermediary mechanisms.

Specifically, exercise self-efficacy functions as an initial cognitive mechanism that translates psychological well-being into behavioral beliefs. In line with Social Cognitive Theory, individuals with higher mental health are more likely to exhibit stronger self-efficacy due to increased motivation, positive affect, and self-regulation capacity [11,12]. However, the relatively modest direct effect of self-efficacy on quality of life suggests that its role is primarily indirect, operating through higher-order adaptive mechanisms. This interpretation is supported by recent evidence indicating that self-efficacy contributes to well-being largely through mediating pathways rather than direct effects [9,60].

Psychological resilience, in contrast, emerged as a stronger predictor of quality of life, highlighting its role as a central adaptive mechanism. Individuals with higher resilience are better able to manage stress and maintain functioning under adverse conditions, particularly in high-stress professions such as oncology nursing [15,20,29]. Recent studies further emphasize that resilience plays a protective role in mitigating burnout and sustaining well-being among healthcare professionals [3].

Importantly, the findings provide strong support for the proposed serial mediation structure. The pathway from mental health to quality of life operates through a coherent sequence: mental health enhances self-efficacy, which in turn strengthens psychological resilience, ultimately leading to improved quality of life. This structure reflects a theoretically grounded progression from emotional resources to cognitive beliefs and finally to adaptive capacity, aligning with Social Cognitive Theory and process-based models of well-being [11,60]. More specifically, within Social Cognitive Theory, self-efficacy serves as a central mechanism through which individuals regulate behavior and cope with environmental demands. In contrast, the Theory of Planned Behavior explains how perceived behavioral control facilitates adaptive responses. The present findings integrate these perspectives by demonstrating how emotional resources (mental health) are translated into cognitive beliefs (self-efficacy) and subsequently into adaptive capacity (resilience), ultimately influencing quality of life. These findings are consistent with prior research indicating that complex behavioral constructs are shaped by multidimensional factors rather than isolated predictors, thereby supporting the use of integrative, process-oriented models in explaining human behavior [10].

Despite these contributions, several limitations should be acknowledged. The cross-sectional design limits causal inference, and the possibility of reciprocal relationships cannot be excluded. Additionally, the use of self-report measures may introduce common method bias, although statistical controls were applied [48]. Future research is encouraged to employ longitudinal and multi-method designs to better establish causal pathways and temporal dynamics.

## 5. Conclusions

This study demonstrates that the impact of mental health on quality of life among oncology nurses is not merely direct but is also shaped by underlying psychosocial mechanisms, particularly exercise self-efficacy and psychological resilience. The findings indicate that mental health functions as a critical starting point. At the same time, its translation into quality of life occurs through the interaction of cognitive and adaptive processes, supporting the multidimensional and dynamic nature of well-being [7,11].

More specifically, exercise self-efficacy appears to operate as a cognitive mechanism that strengthens individuals’ beliefs in their capacity to act. In contrast, psychological resilience serves as an adaptive mechanism that supports coping and sustained functioning under stress. This sequential structure suggests that quality of life is shaped through a progressive pathway in which cognitive confidence is transformed into adaptive capacity and, ultimately, improved well-being [15,20]. This process is particularly relevant for oncology nurses, who are frequently exposed to emotional labor, occupational stress, and recurrent patient loss [1,29].

From a practical perspective, the findings suggest that interventions to improve oncology nurses’ quality of life should not focus solely on mental health. However, they should also strengthen exercise self-efficacy and psychological resilience. Integrated psychosocial programs that target emotional, cognitive, and adaptive resources may provide more sustainable support for healthcare professionals working in high-stress clinical settings [3].

In conclusion, this study contributes to the well-being literature by showing that the relationship between mental health and quality of life operates through a sequential and mechanism-based process. By integrating exercise self-efficacy and psychological resilience within a serial mediation framework, the study offers a more explanatory understanding of how psychological resources are translated into quality-of-life outcomes among oncology nurses.

## 6. Limitations

Despite its contributions, this study has several limitations that should be acknowledged.

First, the cross-sectional design limits the ability to draw causal inferences about the relationships among mental health, exercise self-efficacy, psychological resilience, and quality of life. Although the proposed model is theoretically grounded, the temporal ordering of variables cannot be empirically confirmed [23].

Second, the specified serial mediation structure reflects one theoretically plausible pathway; however, alternative model configurations (e.g., reciprocal or reversed relationships) were not tested, which may limit the model’s comprehensiveness.

Third, the use of self-report measures from a single data source raises the possibility of common method bias and response-related biases [48,61].

Fourth, the sample consisted exclusively of oncology nurses, which may limit the generalizability of the findings to other professional or cultural contexts.

Finally, the study focused solely on individual-level variables and did not include organizational or contextual factors, which may also influence mental health and quality of life.

## 7. Recommendations

### 7.1. Research Implications

Future research should adopt longitudinal and experimental designs to establish causal relationships and examine the temporal dynamics of the proposed model [23]. Additionally, alternative model configurations should be tested using advanced analytical approaches, such as structural equation modeling and cross-lagged panel designs, to evaluate competing pathways. To improve external validity, future studies should examine the model across different healthcare professions and cultural contexts [62]. Incorporating organizational and contextual variables (e.g., burnout, emotional labor, workload, and perceived organizational support) into multi-level frameworks would provide a more comprehensive understanding of quality of life. Finally, intervention-based research is recommended to test whether enhancing self-efficacy and psychological resilience can effectively improve quality of life in practice.

### 7.2. Practical Implications

The findings provide important practical implications for improving the quality of life of oncology nurses. Specifically, the results indicate that mental health influences quality of life not only directly but also through key psychosocial mechanisms, namely exercise self-efficacy and psychological resilience.

Accordingly, interventions should adopt an integrated approach that simultaneously targets emotional, cognitive, and adaptive processes. Programs that promote physical activity, enhance self-efficacy, and develop psychological resilience may be particularly effective in supporting nurses’ well-being.

At the organizational level, improving working conditions, managing workload, and strengthening psychosocial support systems are essential to sustain these individual-level gains.

Overall, combining individual-focused and organizational-level strategies within a multidimensional framework is likely to produce more sustainable improvements in the well-being of healthcare professionals.

### 7.3. Education and Policy Implications

At the policy level, this study’s findings indicate that educational and institutional policies intended to support the well-being of healthcare workers need to be restructured. In particular, rather than approaches that focus solely on improving mental health, developing holistic intervention programs that also address exercise self-efficacy and psychological resilience would be more effective. In this regard, integrating psychosocial support services with practices that promote physical activity in health policies is important.

In terms of education, it is recommended that modules based on stress management, self-efficacy development, and psychological resilience be included in nursing education programs. In addition, in-service training should be structured to include experiential and skills-oriented approaches, rather than just information transfer. Furthermore, collaborative models established among health institutions, universities, and public authorities will play a critical role in disseminating sustainable practices to improve the quality of life of healthcare workers. Such multi-stakeholder policies will both strengthen individual well-being and contribute positively to the overall functioning of the health system. Developing education and public policies within a holistic framework that considers both individual psychological resources and institutional support mechanisms will help sustainably improve the quality of life for healthcare professionals.

## Figures and Tables

**Table 1:** Descriptive statistics of participants.

Variables	Groups	N	(%)
Age	Under 30	238	39.4
	31–40	158	26.2
	41 and above	208	34.4
Gender	Female	561	92.9
	Male	43	7.1
Hospital at work	State	327	54.1
	Private	277	45.9
Economic Status	Low	92	15.2
	Middle	466	77.2
	High	46	7.6
Years of work experience	5–10	305	50.5
	11–20	160	26.5
	21 and above	139	23.0

**Table 2:** Reliability analysis results of the scales.

Scales	Number of Items	Cronbach’s Alpha
Mental Health Continuum	14	0.93
Exercise Self-efficacy	6	0.82
Psychological Resilience	6	0.85
World Health Organization Quality of Life	26	0.95

**Table 3:** Descriptive statistics of scale scores.

Scales	Min.	Max.	X ± SS	Skewness	Kurtosis
Mental Health Continuum	6.00	50.00	28.32 ± 13.04	−0.00	−1.10
Exercise Self-efficacy	7.00	28.00	14.53 ± 3.93	0.19	−0.53
Psychological Resilience	6.00	25.00	17.88 ± 4.76	−0.27	−0.80
Quality of Life	41.00	118.00	82.87 ± 19.33	−0.19	−0.78

N = 604.

**Table 4:** Correlation analysis between nurses’ mental health continuum, exercise self-efficacy, psychological resilience, and quality of life.

Scales	1	2	3	4
Mental Health Continuum	1	0.26 **	0.24 **	0.58 **
Exercise Self-efficacy	0.26 **	1	0.46 **	0.47 **
Psychological Resilience	0.24 **	0.46 **	1	0.58 **
Quality of Life	0.58 **	0.47 **	0.58 **	1

Note: ** *p* < 0.01. 1 = Mental Health Continuum; 2 = Exercise Self-efficacy; 3 = Psychological Resilience; 4 = Quality of Life.

**Table 5:** The effect of the mental health continuum on quality of life.

Variables									
Independent	Depend	β	SE	t	*p*	R	R^2^	F	*p*
Mental Health Continuum	Quality of Life	0.86	0.05	17.33	0.000	0.58	0.33	300.43	0.000 **

N = 604; ** *p* < 0.001.

**Table 6:** Collinearity statistics of predictor variables.

Model	Collinearity Statistics	
1	Tolerance	VIF
Exercise Self-efficacy	0.768	1.302
Psychological Resilience	0.775	1.291
Mental Health Continuum	0.915	1.092

**Table 7:** Cook’s distance statistics.

Statistic	Min.	Max.	Mean	SD	N
Cook’s Distance	0.000	0.088	0.003	0.010	604

**Table 8:** Regression model summary.

Model	R	R^2^	Adjusted R^2^	Std. Error	F	*p*
1	0.751	0.564	0.562	12.7976	258.575	0.000 **

** *p* < 0.01.

**Table 9:** Analysis of variance (ANOVA) for the regression model.

Model		Sum of Squares	df	Mean Square	F	*p*
	Regression	127,047.420	3	42,349.140		
1	Residual	98,267.247	600	163.779	258.575	0.000 **
	Total	225,314.667	603			

** *p* < 0.01.

**Table 10:** Regression Coefficients for Predictors of Quality of Life.

Model	Unstandardized Coefficients	Standardized Coefficients			
1	B	Std. Error	β	t	*p*
Exercise Self-efficacy	0.878	0.151	0.178	5.795	0.000
psychological Resilience	1.597	0.124	0.393	12.836	0.000
Mental Health Continuum	0.648	0.042	0.437	15.510	0.000

** *p* < 0.01.

**Table 11:** The Serial Mediation Role of Exercise Self-efficacy and Psychological Resilience between Mental Health Continuum and Quality of Life (N = 604).

Outcomes												
	Exercise Self-Efficacy (M1)				Psychological Resilience(M2)				Quality of Life (Y)			
		b	SE	t		b	SE	t		b	SE	t
Mental Health Continuum (X)	a1	0.08	0.01	6.50	a2	0.05	0.01	3.53	c′	0.65	0.04	15.51
Exercise Self-efficacy (M1)	-	-	-	-	d1	0.51	0.05	11.41	b1	0.88	0.15	5.80
Psychological Resilience (M2)	-	-	-	-	-	-	-	-	b2	1.60	0.12	12.84
Constant												
		R^2^ = 0.06				R^2^ = 0.22				R^2^ = 0.56		
		F(1, 602) = 42.28				F(1, 602) = 87.44				F(1, 602) = 258.58		
		*p* = 0.000 **				*p* = 0.000 **				*p* = 0.000 **		

** *p* < 0.01.

**Table 12:** The Indirect Effects of Mental Health Continuum and Quality of Life.

Indirect Effects	b	SE	LLCI	ULCI
Total effect	0.21	0.04	0.13	0.30
Ind 1	0.07	0.02	0.03	0.11
Ind 2	0.08	0.03	0.03	0.15
Ind 3	0.06	0.02	0.04	0.10

Note: Ind = indirect effect. Ind1 = Mental Health Continuum → Exercise Self-efficacy → Quality of Life; Ind2 = Mental Health Continuum → Psychological Resilience → Quality of Life; nd3 = Mental Health Continuum → Exercise Self-efficacy → Psychological Resilience → Quality of Life. LLCI = Lower Level Confidence Interval; ULCI = Upper Level Confidence Interval. Indirect effects are considered statistically significant when the 95% confidence interval does not include zero.

## Data Availability

The data presented in this study are available upon request from the corresponding author due to ethical and privacy restrictions. The dataset contains sensitive personal and clinical information, and participant consent does not allow public sharing.
